# A novel allele of L-galactono-1,4-lactone dehydrogenase is associated with enhanced drought tolerance through affecting stomatal aperture in common wheat

**DOI:** 10.1038/srep30177

**Published:** 2016-07-22

**Authors:** Juncheng Zhang, Bin Li, Yanping Yang, Peiyuan Mu, Weiqiang Qian, Lingli Dong, Kunpu Zhang, Xin Liu, Huanju Qin, Hongqing Ling, Daowen Wang

**Affiliations:** 1 The State Key Laboratory of Plant Cell and Chromosome Engineering, Institute of Genetics and Developmental Biology, Chinese Academy of Sciences, Beijing 100101, China; 2 University of Chinese Academy of Sciences, Beijing 100049, China; 3 The National Key Facility for Crop Gene Resources and Genetic Improvement, Institute of Crop Science, Chinese Academy of Agricultural Sciences, Beijing 100081, China; 4 Institute of Crop Research, Xinjiang Academy of Agri-Reclamation Sciences, Shihezi 832000, China; 5 The Collaborative Innovation Center for Grain Crops, Henan Agricultural University, Zhengzhou 450002, China

## Abstract

In higher plants, L-galactono-1,4-lactone dehydrogenase (GLDH) plays important roles in ascorbic acid (AsA) biosynthesis and assembly of respiration complex I. Here we report three homoeologous genes (*TaGLDH-A1*, -*B1* and -*D1*) encoding common wheat GLDH isozymes and a unique allelic variant (*TaGLDH-A1b*) associated with enhanced drought tolerance. *TaGLDH-A1*, -*B1* and -*D1* were located on chromosomes 5A, 5B and 5D, respectively, and their transcripts were found in multiple organs. The three homoeologs each conferred increased GLDH activity when ectopically expressed in tobacco. Decreasing *TaGLDH* expression in wheat significantly reduced GLDH activity and AsA content. *TaGLDH-A1b* differed from wild type allele *TaGLDH-A1a* by an in-frame deletion of three nucleotides. TaGLDH-A1b was biochemically less active than TaGLDH-A1a, and the total GLDH activity levels were generally lower in the cultivars carrying *TaGLDH-A1b* relative to those with *TaGLDH-A1a*. Interestingly, *TaGLDH-A1b* cultivars showed stronger water deficiency tolerance than *TaGLDH-A1a* cultivars, and *TaGLDH-A1b* co-segregated with decreased leaf water loss in a F_2_ population. Finally, *TaGLDH-A1b* cultivars generally exhibited smaller leaf stomatal aperture than *TaGLDH-A1a* varieties in control or water deficiency environments. Our work provides new information on *GLDH* genes and function in higher plants. *TaGLDH-A1b* is likely useful for further studying and improving wheat tolerance to drought stress.

Drought is a major abiotic stress decreasing global crop production and food security^1^,^2^. It may become even more severe owing to global warming^3^. Consequently, understanding and improving crop tolerance to drought is a top target in current plant biology and breeding research^1^,^2^,^4^,^5^. Drought tolerance is a complex trait controlled by polygenes, involving multiple physiological and developmental processes and affected by the environment^1^,^2^. The advent of molecular genetic and functional genomic studies has substantially improved understanding on drought response in model plants (e.g., *Arabidopsis thaliana* and rice)^5^,^6^,^7^. However, much less is known about the genetic and molecular basis of drought tolerance in crop species^1^,^2^. This is particularly evident for common wheat (*Triticum aestivum*, 2n = 6x = 42, AABBDD), which carries a large and complex hexaploid genome and has only limited functional genomic resources for efficiently studying the molecular basis of drought tolerance and other agronomic traits^8^,^9^.

L-galactono-1,4-lactone dehydrogenase (GLDH) (EC 1.3.2.3) is a mitochondrion located enzyme found in many eukaryotic organisms^10^,^11^,^12^,^13^. In higher plants, GLDH is essential for the synthesis of ascorbic acid (AsA), a vital and abundant antioxidant, through the D-mannose/L-galactose pathway^14^. GLDH acts in the final step of this pathway, converting L-galactono-1,4-lactone (L-GalL) into AsA^14^. GLDH has also been found required for the assembly and accumulation of plant respiratory complex I (RCI)^10^,^15^. Through transgenic studies in multiple plant species, GLDH activities have been shown required for the normal growth and development of plant cells and organs and their efficient response to adverse environmental factors. For example, in both tobacco and tomato, suppression of GLDH function results in decreased cell and organ growth^16^,^17^. In rice, inhibition of GLDH activity leads to decreased AsA content, with the transgenic plants exhibiting reductions in seed set and tiller number^18^,^19^. In *Arabidopsis*, our study revealed that partial reduction of GLDH expression through RNA interference not only lowers AsA content, but also causes a substantial decrease of foliar water loss rate and potential tolerance to drought stress^20^. Overexpression of GLDH confers enhanced tolerance to oxidative stress in tobacco cells^21^ and to salt stress in tobacco plants^22^. In the studies above, changes in GLDH expression and activity levels are mainly rendered using transgenic technology. To date, there is still no published information on natural molecular variations of *GLDH* genes and their potential influences on agronomic traits. One study has revealed that GLDH protein and activity levels differ significantly between two common wheat cultivars with high (cv Buck Chambergo, BCH) or low (cv Cooperativa Maipún, CM) AsA content, and that drought treatment can substantially up-regulate the amounts of GLDH protein and activity in CM^13^. However, the molecular basis underlying GLDH expression difference between the two varieties is still unclear, and the functional importance of such difference in wheat drought tolerance requires further study.

The findings described by Bartoli and colleagues^13^ and the observation that partial suppression of *Arabidopsis* GLDH activity led to enhanced drought tolerance^20^ stimulated us to further study the potential involvement of GLDH in common wheat response to water deficiency stress. Towards this end, we characterized the three homoeologous genes (designated *TaGLDH-A1*, *-B1* and *-D1*, respectively) encoding common wheat GLDH isozymes, tested their GLDH activity through viral vector mediated ectopic expression in *Nicotiana benthamiana*, analyzed their function in AsA biosynthesis using virus induced gene silencing (VIGS), and examined their molecular variations in common wheat germplasm lines. On the basis of these experiments, we comparatively analyzed two alleles of *TaGLDH-A1*, the major wild type (WT) allele *TaGLDH-A1a* and a rare allele *TaGLDH-A1b*, in more detail, and found that *TaGLDH-A1b* was associated with the enhanced drought tolerance of a number of varieties cultivated in arid regions. Our work provides new information on GLDH encoding genes and their function in a polyploid crop species, and demonstrates the association between GLDH and plant tolerance to drought stress.

## Results

### Characteristics of *TaGLDH-A1*, *-B1* and *-D1* homoeologs

**Figure 1:**
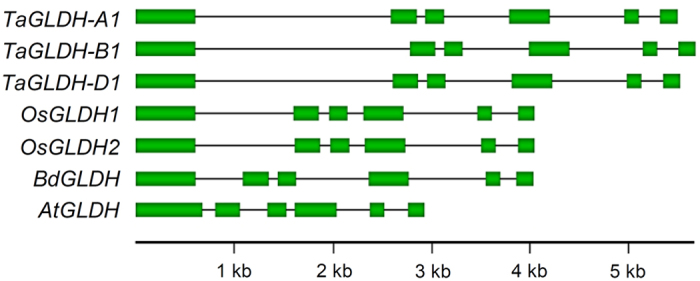
Conservation of exon and intron structure among *TaGLDH-A1*, *-B1* and *-D1* homoeologs and orthologous *GLDH* genes in rice (*OsGLDH1* and *OsGLDH2*), *B.* *distachyon* (*BdGLDH*) and *Arabidopsis* (*AtGLDH*). The chromosomal loci of *OsGLDH1*, *OsGLDH2*, *BdGLDH* and *AtGLDH* are *Os11g0143500*, *Os12g0139600*, *Bradi4g43070* and *At3g47930*, respectively. The GenBank accession numbers for *TaGLDH-A1*, *-B1* and *-D1* are KU695146, KU695147 and KU695148, respectively.

**Figure 2:**
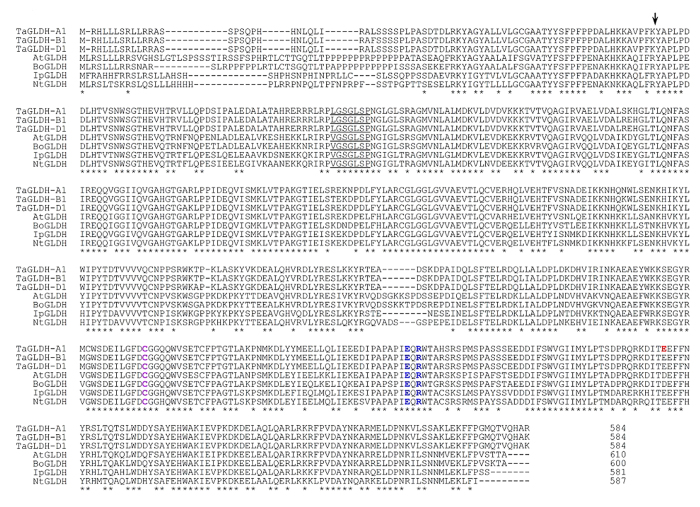
Multiple alignment of the deduced amino acid sequences of TaGLDH-A1, -B1 and -D1 homoeologs and orthologous GLDH proteins from *Arabidopsis* (AtGLDH), cauliflower (BoGLDH), sweet potato (IbGLDH) and tobacco (NtGLDH). Arrow indicates the likely cleavage site of the mitochondrial targeting peptide. The underlined sequence element is required for FAD binding. The conserved cysteine residue labeled in purple is involved in regulating the enzyme activity of plant GLDH proteins, whereas the E-R pair of residues marked in blue are required for efficient substrate binding and catalysis. The glutamic acid (E) marked in red is deleted in a novel allele (TaGLDH-A1b) of TaGLDH-A1. Asterisks denote residues conserved among the compared GLDHs. The UniProtKB numbers for AtGLDH, BoGLDH, IbGLDH and NtGLDH are Q8GY16, O47881, Q9ZWJ1 and Q9FXL9, respectively.

To investigate the genes encoding TaGLDH-A1, -B1 and -D1 isozymes in common wheat, a bacterial artificial chromosome (BAC) library prepared for the bread wheat variety Xiaoyan 54 was screened by PCR (see Methods). A total of seven unique positive BAC clones containing *TaGLDH* sequence were identified. Three similar but not identical *TaGLDH* genomic open reading frames (ORFs) were amplified from the seven BAC clones, and completely sequenced. The genes represented by the three ORFs were designated as *TaGLDH-A1*, *-B1* and *-D1* based on their chromosomal locations (see below). The complete genomic ORF of *TaGLDH-A1*, *-B1* and *-D1* (from start to stop codons) was 5498, 5685 or 5526 bp, and each was composed of six exons and five introns (Fig. 1). This exon-intron pattern was also found for the *GLDH* genes in other monocot (e.g., rice and *Brachypodium distachyon*) and dicot (e.g., *Arabidopsis thaliana*) plants (Fig. 1). The ORF sequences of *TaGLDH-A1*, *-B1* and *-D1* were 97% identical, with nucleotide differences found mainly in the introns. The coding region sequences of *TaGLDH-A1*, *-B1* and *-D1*, as deduced from their cDNA clones, were all 1752 bp (excluding the stop codon) and 99% identical. The deduced proteins of the three homoeologs all contained 584 residues, and were 98–99% identical. Comparison with previously characterized GLDH proteins from *Arabidopsis*, cauliflower, sweet potato and tobacco indicated the presence of a mitochondrial targeting signal peptide (78 residues) and conservation of the residues involved in FAD-binding and those taking part in substrate recognition and catalysis in TaGLDH-A1, -B1 and -D1 (Fig. 2). The calculated molecular mass was 66.1 kD for TaGLDH-A1, -B1 and -D1 preproteins, and 57.5 kD for their putative mature proteins (after removing the putative mitochondrial targeting signal peptide).

To find the chromosome locations of *TaGLDH-A1*, *-B1* and *-D1*, PCR mapping was carried out using the amplicons yielded by primer sets PS3 and PS4 (Table S1, Figure S1). In the PCR with the genomic DNA of Xiaoyan 54 or Chinese Spring (CS) as templates, both PS3 and PS4 gave rise to three amplicons with variable size diagnostic for *TaGLDH-A1*, *-B1* and *-D1* (Figure S2). Through examining amplicons from the nulli-tetrasomic (NT) lines of CS^23^, *TaGLDH-A1*, *-B1* and *-D1* were assigned to 5A, 5B and 5D chromosomes, respectively (Figure S2). Consistent with this finding, three different *GLDH* genes (*Traes_5AL_E7806F3C1*, *Traes_5BL_E8D36EEA8* and *Traes_5DL_696F6D975*) have been annotated in the draft genome sequence of CS^24^. These three genes are located on the long arms of 5A, 5B and 5D, respectively. The deduced amino acid sequences of *Traes_5BL_E8D36EEA8* and *Traes_5DL_696F6D975* are identical to those of TaGLDH-B1 and -D1, respectively. However, the gene model annotated for *Traes_5AL_E7806F3C1* is partial because the polypeptide deduced from it contains only 400 amino acids. Nevertheless, this hypothetical polypeptide is nearly identical to the last 400 residues of TaGLDH-A1.

### Expression pattern of *TaGLDH* in common wheat organs

**Figure 3:**
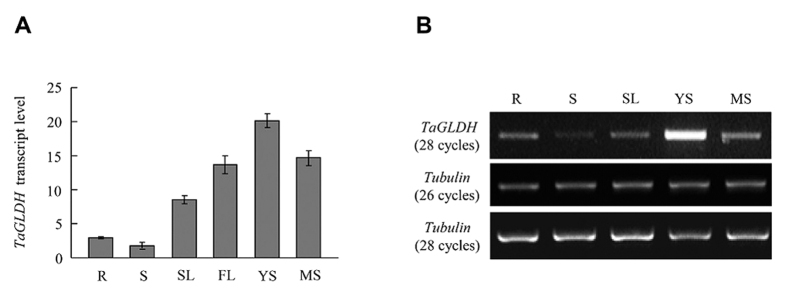
Analysis of relative transcript levels of *TaGLDH* in different common wheat organs using qRT-PCR (**A**) and semi-quantitative RT-PCR (**B**). R: root; S: stem; SL: seedling leaf; FL: flag leaf; YS: young spikelet; MS: mature spikelet. The values shown in (**A**) are means ± SD of three biological replicates. In (**B**), a wheat *tubulin* gene (GenBank accession U76745) was used as the internal control. Three separate semi-quantitative RT-PCR assays were conducted with highly similar results obtained.

As revealed by qRT-PCR (Fig. 3A), the expression level of *TaGLDH* was highest in the young spikelets, intermediate in the seedling leaves, flag leaves and mature spikelets, and comparatively low in the roots and stems. The relatively high expression level of *TaGLDH* in young spikelets was also found by semi-quantitative RT-PCR assay comparing *TaGLDH* transcript levels in five vegetative and reproductive organs of common wheat (Fig. 3B).

### GLDH activity of TaGLDH-A1, -B1 and -D1

**Figure 4:**
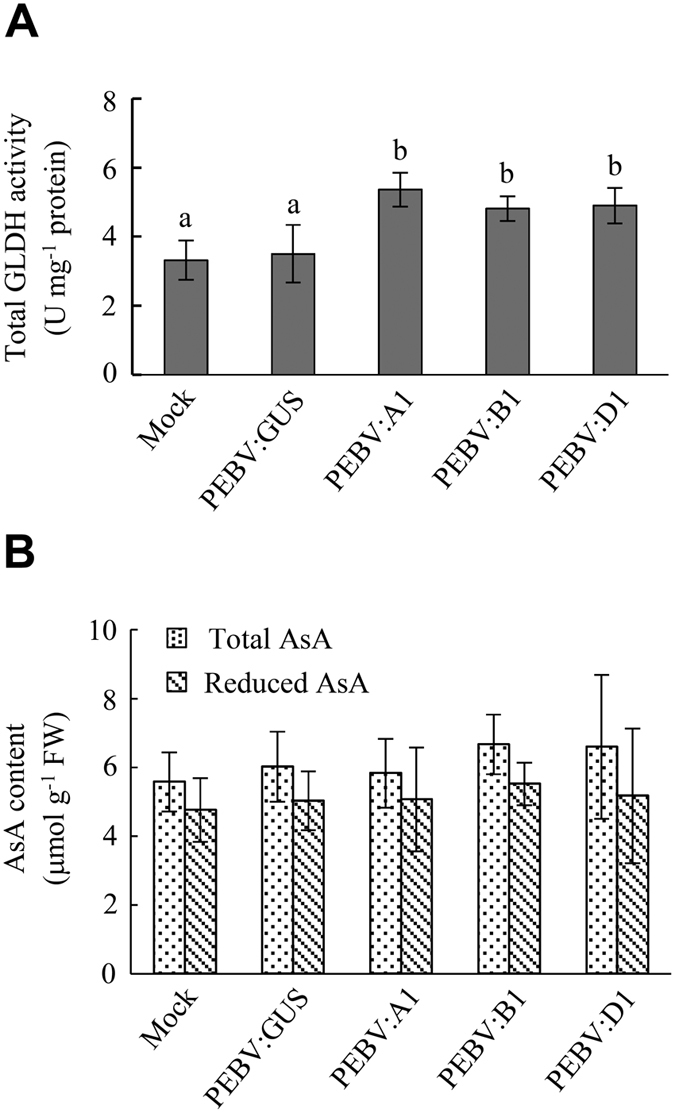
Ectopic expression of *TaGLDH-A1*, *-B1* and *-D1* homoeologs in *N. benthamiana* using PEBV viral vector. PEBV:A1, PEBV:B1 and PEBV:D1 are three recombinant viruses expressing TaGLDH-A1, -B1 and -D1, respectively. PEBV:GUS is another recombinant virus expressing a bacterial *GUS* gene (encoding β-glucuronidase). (**A**) Total GLDH activity levels in mock controls and the plants infected by different recombinant viruses. The values displayed are means ± SD of three measurements for three different plants. Different letters above the histograms indicate statistical difference (*P* < 0.05). (**B**) Total and reduced AsA contents in mock controls and the plants infected by different recombinant viruses. The contents shown are means ± SD of three measurements of three different plants. Two independent experiments were performed with similar results obtained.

To examine if TaGLDH-A1, -B1 and -D1 may possess GLDH activity, they were individually expressed in *N. benthamiana* using the viral vector based on pea early browning virus (PEBV, see Methods). Four recombinant viruses (PEBV:GUS, PEBV:A1, PEBV:B1 and PEBV:D1, Figure S3a) were constructed and introduced into *N. benthamiana* plants. Two weeks after the inoculation of PEBV:GUS, abundant β-glucuronidase (GUS) staining signals were detected in the inoculated leaves (Figure S3b), thus confirming the capacity of PEBV vector to mediate high level of ectopic expression of heterologous coding sequence in *N. benthamiana*. Subsequently, the inoculated leaves in the plants infected by PEBV:GUS, PEBV:A1, PEBV:B1 or PEBV:D1 were collected and examined for total GLDH enzyme activity. Additionally, the mock-inoculated leaves in the control plants were also analyzed for GLDH activity. From Fig. 4A, it is clear that GLDH activity level was significantly higher in the leaves infected by PEBV:A1, PEBV:B1 or PEBV:D1 than in those infected by PEBV:GUS, but there was no significant difference in GLDH activity level between PEBV:GUS infected leaves and the mock-inoculated controls. However, the total AsA content did not differ significantly among the examined leaf samples (Fig. 4B).

### Function of *TaGLDH* in AsA biosynthesis

To investigate the involvement of *TaGLDH* in AsA biosynthesis in common wheat, the effects of decreasing *TaGLDH* expression on AsA content were analyzed. The reduction of *TaGLDH* expression was achieved by VIGS with the vector derived from barley stripe mosaic virus (BSMV) (see Methods). Among the three recombinant BSMVs (BSMV:GFP, BSMV:PDSas and BSMV:GLDHas, Figure S4a) used in this analysis, BSMV:GLDHas was developed to specifically silence all three *TaGLDH* homoeologs in common wheat. A 431 bp sequence element strictly conserved among *TaGLDH-A1*, *-B1* and *-D1* was employed to construct BSMV:GLDHas. This element recognized only the three *GLDH* genes (*Traes_5AL_E7806F3C1*, *Traes_5BL_E8D36EEA8* and *Traes_5DL_696F6D975*, see above) in the draft genome sequence of CS. BSMV:GFP expresses green fluorescent protein (GFP) in wheat cells, and is useful for monitoring virus spread in the inoculated seedlings. BSMV:PDSas, silencing wheat phytoene desaturase (PDS) gene and causing leaf bleaching, is valuable for checking the progress of VIGS.

**Figure 5:**
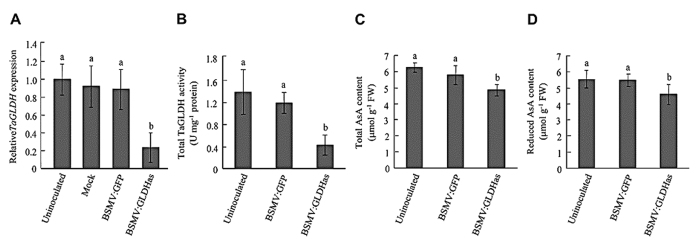
Function of *TaGLDH* in AsA biosynthesis as investigated using BSMV mediated VIGS. BSMV:GFP, expressing a green fluorescence protein, was used for monitoring viral spread in infected wheat plants, whereas BSMV:GLDHas was for silencing *TaGLDH* transcripts. (**A**) Comparison of relative *TaGLDH* expression levels among different plant samples, with *TaGLDH* expression in the uninoculated plants set as 1. *TaGLDH* expression level was assayed by qRT-PCR. Significant decrease of *TaGLDH* expression was detected in the plants infected by BSMV:GLDHas but not in the mock controls or those infected by BSMV:GFP. (**B–D**) Significant reductions of TaGLDH activity and the contents of total and reduced AsA in the plants infected by BSMV:GLDHas but not in those infected by BSMV:GFP. The values depicted are all means ± SD of three measurements for three different plants. Different letters above the histograms mark statistical difference (*P* < 0.05). The assays were repeated at least twice with similar results found.

Two weeks after virus inoculation, strong GFP fluorescence was observed in the leaf tissues of the seedlings infected with BSMV:GFP (Figure S4b), and apparent leaf bleaching appeared in those infected by BSMV:PDSas (Figure S4c), thus verifying the effectiveness of BSMV VIGS in this work. In the seedlings infected by BSMV:GLDHas, the expression level of *TaGLDH* was significantly reduced (by >75%) relative to that detected for the controls (uninoculated, mock-inoculated or BSMV:GFP infected seedlings, Fig. 5A). In line with this finding, total TaGLDH activity level in BSMV:GLDHas-infected seedlings was substantially decreased (by >70%) relative to that in the controls (mock-inoculated or infected by BSMV:GFP, Fig. 5B). Silencing *TaGLDH* expression caused significant reductions of total AsA (Fig. 5C) and reduced AsA (Fig. 5D) contents in the plants infected by BSMV:GLDHas, but for both parameters the reduction was only about 20%.

Past studies have shown that exogenous application of L-GalL can substantially stimulate AsA biosynthesis in plant tissues^25^,^26^. Thus, the small decrease of AsA content in the plants infected by BSMV:GLDHas might be due to an insufficient amount of L-GalL available for AsA biosynthesis in the cells. To investigate this possibility, we exogenously applied 15 mM L-GalL to the leaf segments excised from BSMV:GLDHas infected seedlings and the controls, followed by measuring total and reduced AsA contents. For the controls (mock-inoculated or infected with BSMV:GFP), the leaf segments treated with L-GalL exhibited dramatically increased total and reduced AsA contents (by 6.32–6.63 times) relative to those without exogenous application of the substrate (Figure S5). For the plants infected by BSMV:GLDHas, treatment with L-GalL also resulted in increases of total and reduced AsA contents but at much lower levels (by 3.83–3.98 times, Figure S5). In the presence of 15 mM L-GalL, the total and reduced AsA contents in BSMV:GLDHas infected leaf tissues were reduced by about 42% relative to those of the controls (Figure S5). This level of reduction was substantially higher than that found for the BSMV:GLDHas infected leaf tissues (~20%, Fig. 5C,D) in the absence of artificially supplied L-GalL.

### Identification and characterization of *TaGLDH-A1b*

Potential molecular variations in the coding region of *TaGLDH-A1*, *-B1* or *-D1* in the micro-core collection (MCC) of Chinese wheat germplasm were examined by genomic PCR with the primer sets PS3 and PS4 (Table S1, Figure S1), respectively. The 262 MCC lines, including both land races and improved varieties, represent 70% of the genetic diversity of Chinese common wheat^27^. No length variation was detected in the amplicons produced by PS3. However, in the amplicons by PS4, length variation was found for *TaGLDH-A1* in four unique cultivars (i.e., Hongdongmai, Kashi 1, Kashibaipi and Tutoumai, Figure S6). The size of *TaGLDH-A1* amplicons in the four lines was 452 bp, which was 3 bp shorter than those from Xiaoyan 54 and the remaining MCC lines (Figure S6). Sequencing the full-length genomic ORF and cDNA coding region of *TaGLDH-A1* in the four cultivars revealed an in-frame deletion of three nucleotides (GAG) compared to *TaGLDH-A1* sequence in Xiaoyan 54. However, the four lines did not differ from Xiaoyan 54 in either the coding sequence of *TaGLDH-B1* or that of *TaGLDH-D1*. To facilitate further characterization, the *TaGLDH-A1* sequence in Xiaoyan 54 was designated as the WT allele *TaGLDH-A1a*, whereas that in Hongdongmai, Kashi 1, Kashibaipi and Tutoumai was named as *TaGLDH-A1b*. Comparison of deduced proteins showed that the glutamic acid residue at position 501 of TaGLDH-A1a was absent in TaGLDH-A1b (Fig. 2). This glutamic acid was 42 residues away from the conserved Glu-Arg pair (Fig. 2). These two residues have been suggested to reside in the active site of plant GLDH proteins, and are thus essential for optimal function of these enzymes^28^.

**Figure 6:**
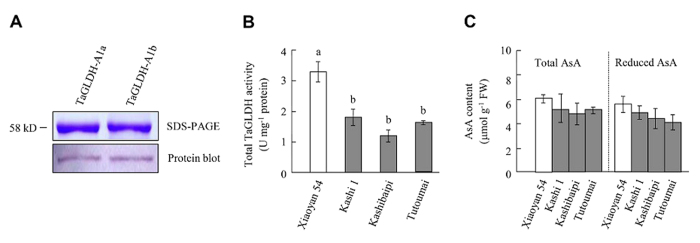
Analysis of TaGLDH-A1a and TaGLDH-A1b alleles. (**A**) SDS-PAGE and protein blot assays of TaGLDH-A1a and TaGLDH-A1b recombinant proteins. The two alleles were expressed in *E. coli* cells without the putative mitochondrial targeting peptide but with a histidine tag at the N-terminal end, followed by purification with nickel affinity chromatography and the protein blot assay using an antibody specific for histidine tag. (**B,C**) Comparison of total TaGLDH activity levels and the contents of total and reduced AsA in Xiaoyan 54 (carrying *TaGLDH-A1a*) and the three Xinjiang wheat cultivars (Kashi 1, Kashibaipi and Tutoumai, with *TaGLDH-A1b*). The values are means ± SD of three measurements for three different plants. Different letters above the histograms indicate statistical difference (*P* < 0.05). For both (**B,C**), the data displayed are typical of three independent assays.

Potential biochemical differences between the proteins specified by *TaGLDH-A1a* or *TaGLDH-A1b* were examined. *TaGLDH-A1a* and *TaGLDH-A1b*, lacking the sequence coding for the putative mitochondrion targeting peptide, were individually expressed in *Escherichia coli* using the bacterial expression vector pET-30a (see Methods), with the aim to produce recombinant TaGLDH-A1a and TaGLDH-A1b proteins without the mitochondrial targeting peptide but with a histidine tag at the N-terminus. The recombinant proteins were purified, and verified by protein blot analysis with an antibody specific for the histidine tag (Fig. 6A). Subsequently, the kinetic properties of purified TaGLDH-A1a and TaGLDH-A1b were investigated using L-GalL as a substrate. Based on their Michaelis-Menten kinetic characteristics, the mean *K*_m_ and *V*_max_ of TaGLDH-A1a were estimated to be 61 μM and 11.43 μmol. mg^−1^ protein.min^−1^, respectively, and the corresponding values for TaGLDH-A1b were 181 μM and 0.16 μmol.mg^−1^protein.min^−1^, respectively (Table 1). These data indicated that TaGLDH-A1b had significantly reduced kinetic activities than TaGLDH-A1a towards the substrate tested. In line with the biochemical difference between TaGLDH-A1a and TaGLDH-A1b, total TaGLDH activity levels in the *TaGLDH-A1b* lines were generally and significantly lower than that found for Xiaoyan 54 carrying *TaGLDH-A1a* (Fig. 6B). Under normal growth conditions, total AsA contents in the seedling leaves of the three *TaGLDH-A1b* lines tended to be lower than that of Xiaoyan 54, but the differences were not statistically significant (Fig. 6C).

### Association of *TaGLDH-A1b* with enhanced tolerance to water deficiency stress

The four cultivars carrying *TaGLDH-A1b* all came from the Xinjiang Uygur Autonomous Region (Xinjiang hereafter) in Northwest China^29^. The common wheat lines cultivated in Xinjiang generally have enhanced drought tolerance because this region has a strong continental climate with an annual rainfall varying from about 40 mm (in the south) to 192 mm (towards the north)^29^,^30^. Therefore, we tested if *TaGLDH-A1b* might be found in an additional set of 11 wheat cultivars from Xinjiang (Table S2) with a newly developed molecular marker CAP-A1b (Figure S7a). The PCR fragments derived from *TaGLDH-A1a* (538 bp) and *TaGLDH-A1b* (535 bp) were digested using the restriction enzyme *Bsa*JI, which cleaved the amplicon of *TaGLDH-A1a* once (yielding two fragments of 266 and 272 bp, respectively) but not that of *TaGLDH-A1b* (Figure S7a). Among the 11 cultivars, three lines (Jiuchun 1, Redstar and Xinchun 3) were detected to have *TaGLDH-A1b*, with the remaining ones carrying *TaGLDH-A1a* (Table S2). Together, seven cultivars carrying *TaGLDH-A1b* were found among a total of 273 germplasm lines examined in this study. The frequency of *TaGLDH-A1b* was thus 2.56%. The seven *TaGLDH-A1b* lines are all known to have enhanced tolerance to abiotic stresses (Table S3), and four of them (Hongdongmai, Kashibaipi, Redstar, and Xinchun 3) have been extensively cultivated in Xinjiang^29^. One of the *TaGLDH-A1b* lines, Redstar, was originally from the former Soviet Union^29^, whereas the remaining ones were native to Xinjiang (Table S3).

**Figure 7:**
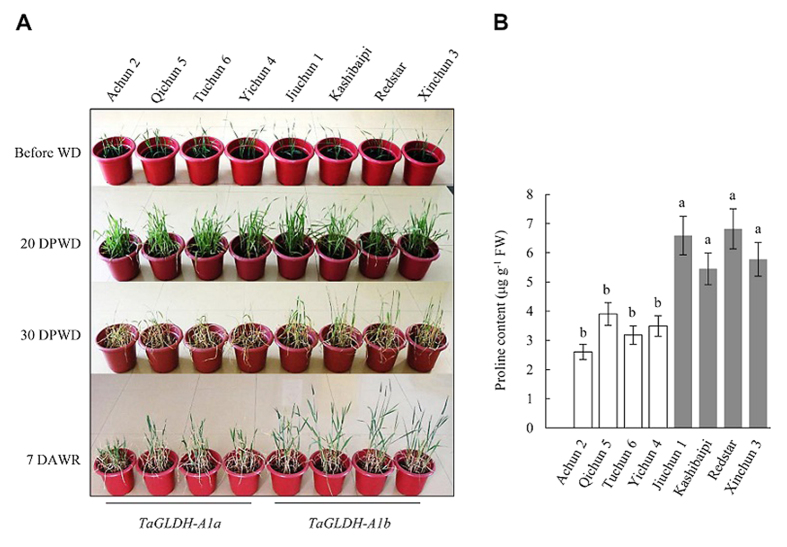
Different responses to water deprivation between the wheat cultivars carrying *TaGLDH-A1a* or *TaGLDH-A1b*. Among the eight Xinjiang wheat cultivars tested, Achun 2, Qichun 5, Tuchun 6 and Yichun 4 had *TaGLDH-A1a* whereas Jiuchun 1, Kashibaipi, Redstar and Xinchun 3 possessed *TaGLDH-A1b*. (**A**) Growth performance of the two types of Xinjiang wheat cultivars before water deprivation (WD), at 20 and 30 days post water deprivation (DPWD), and at 7 days after water resumption (DAWR). The data shown are typical of three separate growth trials. (**B**) Free proline contents in the leaf tissues of the eight cultivars at 20 DPWD. Each value is the mean ± SD of five measurements using five different plants. Different letters above the histograms indicate statistical difference (*P* < 0.05). The data are typical of two independent assays.

Next, we performed three independent pot trials for comparing the drought tolerance of eight Xinjiang cultivars, four carrying *TaGLDH-A1a* and four with *TaGLDH-A1b*. The plants of the eight lines were deprived of water for 30 days followed by rewatering, with their growth behavior recorded at specific time points. At 20 days post water deprivation (DPWD), the four lines with *TaGLDH-A1b* were apparently greener than those carrying *TaGLDH-A1a* (Fig. 7A). At 30 DPWD (just before rewatering), the four *TaGLDH-A1a* lines exhibited severe wilting, but the wilting was much less severe in the four *TaGLDH-A1b* lines (Fig. 7A). At the 7th day after rewatering, the *TaGLDH-A1b* lines were obviously taller and had more green leaves than the ones with *TaGLDH-A1a* (Fig. 7A), indicating that the former lines recovered from water deficiency stress more efficiently than the latter ones. Free proline content is an important indicator of plant drought tolerance^31^. At 20 DPWD, free proline contents in the leaf tissues of *TaGLDH-A1b* lines were generally and significantly higher than those of *TaGLDH-A1a* lines (Fig. 7B). The plants of the eight cultivars, whether carrying *TaGLDH-A1a* or *TaGLDH-A1b*, all survived under the above drought treatment conditions; no difference was observed in the survival rate between the two types of lines in the three pot trials.

**Figure 8:**
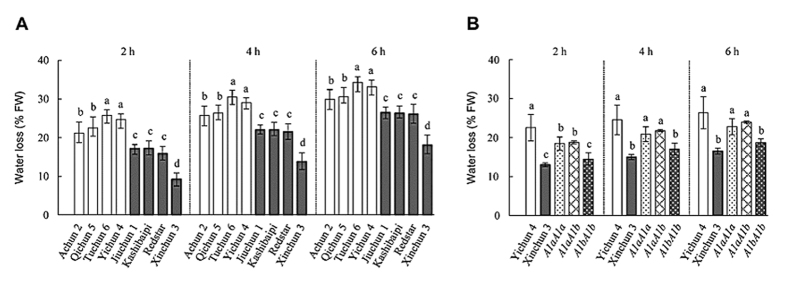
Effects of *TaGLDH-A1a* and *TaGLDH-A1b* on leaf water loss value. Among the eight Xinjiang wheat cultivars used, Achun 2, Qichun 5, Tuchun 6 and Yichun 4 had *TaGLDH-A1a* whereas Jiuchun 1, Kashibaipi, Redstar and Xinchun 3 possessed *TaGLDH-A1b*. (**A**) Water loss values of the eight cultivars determined at 2, 4 and 6 h after leaf detachment, respectively. The four cultivars with *TaGLDH-A1b* exhibited significantly reduced water loss (filled bars) than those carrying *TaGLDH-A1a* (open bars). The data shown are representative of three independent assays. (**B**) Water loss values scored at three different time points after leaf detachment (2, 4 and 6 h) for Yichun 4, Xinchun 3 and the F_2_ seedlings with *A1aA1a* (homozygous for *TaGLDH-A1a*), *A1aA1b* (heterozygous) or *A1bA1b* (homozygous for *TaGLDH-A1b*) genotype. The data depicted are representative of two independent assays. In both (**A,B**), each water loss value is the mean ± SD of three measurements using a pool of eight fully expanded leaves detached from eight separate plants. The histograms labelled by unidentical letters are statistically different (*P* < 0.05).

Lastly, we compared the degree of leaf water loss between *TaGLDH-A1a* and *TaGLDH-A1b* lines because this parameter is another valuable indicator of plant response to water deficiency stress^2^,^5^. Water loss behavior of the leaves was monitored at 2, 4 and 6 h post detachment, respectively, for the two groups of lines carrying *TaGLDH-A1a* or *TaGLDH-A1b*. At all three time points, the levels of water loss recorded for the *TaGLDH-A1b* lines were consistently and significantly lower than those found for the *TaGLDH-A1a* lines (Fig. 8A). Furthermore, we investigated if there would be differences in water loss behavior among the different genotypes identified in a F_2_ population developed using two Xinjiang cultivars Yichun 4 (carrying *TaGLDH-A1a*) and Xinchun 3 (with *TaGLDH-A1b*) (Table S2). After genotyping 134 F_2_ individuals with the DNA marker CAP-A1b (Figure S7b), the individuals homozygous for *TaGLDH-A1a* or *TaGLDH-A1b* were 35 and 30, respectively, and those heterozygous at *TaGLDH-A1* locus were 69. The levels of water loss determined for Xinchun 3 or *TaGLDH-A1b*/*TaGLDH-A1b* leaves were highly similar, both being significantly lower than those obtained for Yichun 4, *TaGLDH-A1a*/*TaGLDH-A1a* or *TaGLDH-A1a*/*TaGLDH-A1b* leaves (Fig. 8B). The water loss levels of Yichun 4, *TaGLDH-A1a*/*TaGLDH-A1a* and *TaGLDH-A1a*/*TaGLDH-A1b* differed only at 2 h post detachment (Fig. 8B).

### Comparison of stomatal aperture between *TaGLDH-A1a* and *TaGLDH-A1b* cultivars

**Figure 9:**
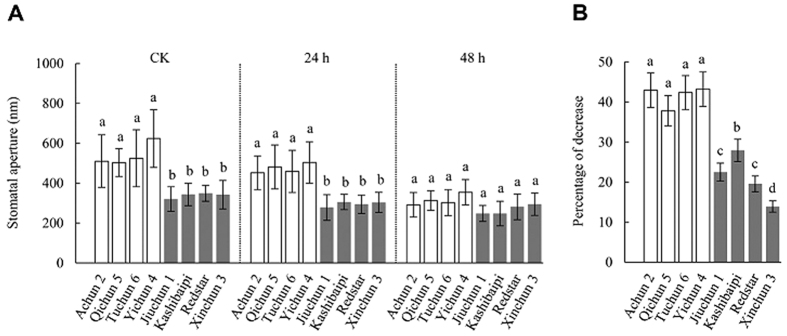
Effects of *TaGLDH-A1a* and *TaGLDH-A1b* on leaf stomatal aperture. Among the eight Xinjiang wheat cultivars examined, Achun 2, Qichun 5, Tuchun 6 and Yichun 4 carried *TaGLDH-A1a* whereas Jiuchun 1, Kashibaipi, Redstar and Xinchun 3 had *TaGLDH-A1b*. (**A**) Stomatal aperture values measured for control plants (CK) and those treated with PEG 6000 for 24 and 48 h, respectively. The four cultivars with *TaGLDH-A1b* showed significantly reduced stomatal aperture (filled bars) than those carrying *TaGLDH-A1a* (open bars) under control conditions and after PEG 6000 treatment for 24 h. Each stomatal aperture value is the mean ± SD of the measurements of at least 200 stomata in six epidermal peels from three plants. The histograms labeled by unidentical letters are statistically different (*P* < 0.05). The data shown are typical of three independent experiments. (**B**) Percentages of stomatal aperture decrease after PEG 6000 treatment for 48 h in the eight cultivars. The percentages were calculated using the data depicted in (**A**). Relative to *TaGLDH-A1a* lines, the extents of stomatal aperture decrease in those with *TaGLDH-A1b* (filled bars) were generally and significantly lower.

The drought tolerance exhibited by *TaGLDH-A1b* cultivars, especially their comparatively lower water loss levels, prompted us to compare stomatal aperture size between *TaGLDH-A1a* and *TaGLDH-A1b* cultivars. Under control conditions, the mean stomatal aperture size of *TaGLDH-A1b* lines (320–349 nm) was substantially lower than that of *TaGLDH-A1a* lines (503–623 nm) (Fig. 9A). Water deficiency stress, imposed by 13% polyethylene glycol 6000 (PEG 6000), caused decreases of stomatal aperture size for both types of lines, with the percentages of the decrease being much stronger for *TaGLDH-A1a* lines (Fig. 9B). After 48 h PEG 6000 treatment, the percentages of stomatal aperture size reduction shown by *TaGLDH-A1b* lines (13.8–27.9%) were generally and significantly lower than those of *TaGLDH-A1a* lines (37.8–43.2%) (Fig. 9B).

## Discussion

Although understanding and enhancing drought tolerance represent a major task in current plant biology and crop improvement research, only a few genes have so far been molecularly characterized for their involvement in wheat drought tolerance^9^,^32^,^33^,^34^ Here, we investigated the function of *TaGLDH* homoeologs and explored the association of a previously undescribed *TaGLDH-A1* allele (*TaGLDH-A1b*) with enhanced drought tolerance by combining molecular, biochemical and physiological approaches. The new information obtained is discussed below.

### Molecular features of *TaGLDH-A1*, *-B1* and *-D1* homoeologs

Prior to this work, little is known about *TaGLDH* homoeologs and their protein products at the molecular level, although there is evidence for TaGLDH protein expression and enzyme activity in common wheat^13^. Based on the data gathered here, several suggestions can be made on the molecular features of *TaGLDH* homoeologs. First, *TaGLDH-A1*, *-B1* and *-D1* are located on wheat group 5 chromosomes. This finding is consistent with the annotation of three *TaGLDH* homoeologous genes on 5A, 5B and 5D chromosomes, respectively, by the draft genome sequence of CS^24^. Second, the exon and intron pattern in *TaGLDH-A1*, *-B1* and *-D1* is identical to that found for orthologous *GLDH* genes in rice, *B. distachyon* and *Arabidopsis*, suggesting the preservation of exon and intron structure in *GLDH* genes in higher plants. Third, the deduced primary structure and mitochondrial targeting of TaGLDH-A1, -B1 and -D1 are highly similar to those of orthologous GLDHs in *Arabidopsis*, cauliflower, sweet potato and tobacco, highlighting the conservation of GLDH protein structure and subcellular location in plant cells. Fourth, TaGLDH-A1, -B1 and -D1 all have GLDH activity, and may not differ substantially from each other in the level of such activity. This is supported by similar increases of total GLDH activity in the *N. benthamiana* plants ectopically expressing *TaGLDH-A1*, *-B1* or *-D1*. Fifth, the recombinant protein of TaGLDH-A1a (lacking the putative mitochondrial targeting peptide) is biochemically active towards the substrate L-GalL, with a *K*_m_ value very similar to that reported for tobacco GLDH enzyme^35^. Lastly, *TaGLDH* transcripts are present in multiple vegetative and reproductive organs of common wheat, with relatively high transcript abundance in the leaf and spike tissues.

The observation of a higher *TaGLDH* transcript level in the leaves by this work agrees well with the finding of abundant TaGLDH proteins in wheat leaf tissues by a previous study^13^. However, it is interesting to note that *TaGLDH* transcripts are highly abundant in the spikelets, particularly in the young spikelet tissues. It is well known that the development of cereal spikelets involves intensive cell growth and differentiation and enhanced metabolic activity^36^, and that the level of reactive oxygen species in this developmental process needs to be tightly controlled especially under stress conditions^37^. Furthermore, AsA has been found to regulate flowering^20^,^38^. Therefore, the high *TaGLDH* transcript level detected in the immature spikelets by this work may reflect the function of TaGLDH in active AsA biosynthesis during common wheat spikelet development. The importance of having sufficient GLDH proteins during cereal spikelet development has recently been demonstrated by the finding that silencing rice *GLDH* expression led to substantially reduced seed set^19^.

### Function of *TaGLDH* in AsA biosynthesis in common wheat

In *Arabidopsis*, rice and several other plant species, the function of *GLDH* in AsA biosynthesis is well supported by genetic evidence obtained using defined mutants and transgenic lines with altered expression *GLDH* level^17^,^19^,^20^,^21^,^22^. But in common wheat genetic evidence for the function of *GLDH* in AsA biosynthesis is not available before this work. From the data of our BSMV VIGS experiment, it is clear that normal *TaGLDH* expression is required for efficient AsA biosynthesis in common wheat seedlings. In agreement with previous studies^39^,^40^,^41^,^42^, our BSMV VIGS was reliable and effectively lowered the expression of *TaGLDH*. However, in the leaf tissues undergoing *TaGLDH* silencing, the reductions of total and reduced AsA contents were considerably below the decreases in *TaGLDH* expression and GLDH activity. The lack of correlation in between *GLDH* expression level, GLDH activity and AsA content has been observed in a number of plant species^13^,^43^, and several possibilities have been suggested to explain such a phenomenon^13^,^25^. Here we found that exogenous application of 15 mM L-GalL strongly increased AsA contents in both controls and the plants infected by BSMV:GLDHas, and substantially enlarged the degree of AsA content reduction by silencing *TaGLDH* expression. Therefore, in common wheat leaf tissues, the function of *TaGLDH* in AsA biosynthesis is at least partly affected by the amount of substrate.

One concern on VIGS data is that their reliability may be affected by off target silencing^39^,^41^. In this work, the chance of off target silencing in the VIGS initiated by BSMV:GLDHas is likely to be very small because the fragment employed for developing BSMV:GLDHas recognized only the three *TaGLDH* homoeologs and no additional *GLDH* paralogous sequences were found in the draft genome sequence of CS. Therefore, the reductions of *TaGLDH* expression, TaGLDH activity and AsA contents in BSMV:GLDHas infected plants are due to specific silencing of the expression of *TaGLDH-A1*, *-B1* and *-D1*. Nevertheless, a more detailed understanding of the function of *TaGLDH-A1*, *-B1* and *-D1* in AsA biosynthesis (and other processes) would require the preparation and examination of wheat mutants lacking one or more of the three homoeologs. We are now in the process of developing such mutants.

### Association of *TaGLDH-A1b* with enhanced drought tolerance

Natural allelic variation is a fundamental mechanism in the genetic control of plant traits^44^. Mining allelic variations at the molecular level has contributed enormously to understanding and improving the genetic basis of valuable traits in higher plants^45^. The cloning of three *TaGLDH* homoeologs in this work enabled us to examine their molecular variations in common wheat germplasm, leading to the discovery of a novel allele *TaGLDH-A1b*. Compared to WT allele (TaGLDH-A1a), TaGLDH-A1b missed one glutamic acid residue, and exhibited a significant reduction in its catalytic activity towards the tested substrate (Table 1). To our knowledge, this type of allele has not been described previously for either plant or animal GLDH proteins. Further, our data suggest that there is very likely a positive association between *TaGLDH-A1b* and enhanced drought tolerance in common wheat. This proposition is supported by four lines of evidence. First, the seven cultivars carrying *TaGLDH-A1b* were all from Xinjiang that has a limited rainfall in wheat production season^29^,^30^. Second, our tests showed that, relative to *TaGLDH-A1a* lines, those with *TaGLDH-A1b* generally displayed better tolerance to water deficiency stress in growth trails (Fig. 7A), had a higher foliar proline content (Fig. 7B), and showed lower leaf water loss rate (Fig. 8A). We did not observe any difference in the survival rate between the cultivars carrying *TaGLDH-A1a* or *TaGLDH-A1b* probably because the drought treatment applied was not sufficiently severe. Third, *TaGLDH-A1b* co-segregated with lower water loss in a F_2_ population (Fig. 8B). Lastly, under either normal or water deficiency conditions, *TaGLDH-A1b* cultivars generally had smaller stomatal aperture (Fig. 9), which is known to contribute directly to reduction of water loss and to stronger drought tolerance in water limited environments^2^,^5^. Interestingly, our previous study showed that partial suppression of GLDH activity also resulted in enhanced drought tolerance in *Arabidopsis*^20^. In both our previous study^20^ and this work, partial reduction of GLDH activity did not cause a drastic decrease of AsA content, and yet drought tolerance was significantly increased. This suggests that, in both monocot and dicot plant species, GLDH activity may be partially lowered to enhance drought tolerance.

Because the frequency of *TaGLDH-A1b* (2.56%) was relatively low, we deduce that *TaGLDH-A1b* is a rare allele that has so far not gained intensive use in wheat breeding. However, *TaGLDH-A1b* may have at least two different geographical origins because one of its carriers (i.e., Redstar) is not native to Xinjiang^29^. There is now growing interest in characterizing rare alleles of plant genes because many of them have been found to confer positive effects on agronomic traits^44^,^45^. For example, a rare allele of the *crtRB1* gene enhances β-carotene content in maize grains^46^. The rare alleles of rice *OsPPKL1* and *GS2/OsGRF4* genes are associated with larger grains and higher yield^47^,^48^. Considering the overwhelming importance of wheat as a major staple food and the urgent need to develop drought tolerant wheat cultivars in both China and the world^9^,^33^,^49^, it is highly worthwhile to test if *TaGLDH-A1b* may confer improved drought tolerance when transferred to high yielding common wheat varieties with more diverse genetic backgrounds. Aided by the CAP-A1b marker developed in this work (Figure S7a), we have now embarked on this test.

### Mechanism underlying the function of *TaGLDH-A1b* in enhanced drought tolerance

From the data gathered in this study (Figs 8 and 9), it is clear that decrease of stomatal aperture and reduction of leaf water loss are two physiological events closely linked with the enhanced drought tolerance by *TaGLDH-A1b*. However, further efforts are needed to reveal the additional physiological processes and molecular interactions involved. A previous study has shown that reducing AsA content in transgenic tobacco plants led to enhanced water stress tolerance through accumulation of a higher concentration of H_2_O_2_ in the guard cells and an increased stomatal closure in water limiting environment^50^. But in common wheat water deficiency treatment, although increasing GLDH activity in certain cultivar, did not cause substantial changes in AsA content^13^. We also found that water deficiency stress did not alter AsA content considerably in the common wheat plants carrying *TaGLDH-A1b* (Figure S8). Furthermore, although the cultivars carrying *TaGLDH-A1b* generally exhibited substantial reductions in TaGLDH activity, their foliar AsA contents did not differ significantly from those of the cultivars with *TaGLDH-A1a* (Fig. 6C). These findings suggest that the overall foliar AsA content may not be directly related to the smaller stomatal aperture observed for *TaGLDH-A1b* plants. However, there may be a difference in the guard cell AsA content between *TaGLDH-A1a* and *TaGLDH-A1b* plants. This is possible because H_2_O_2_ accumulation in the guard cells has been found vital for stomata closing^51^,^52^, and AsA, being the most abundant water-soluble antioxidant in plant cells, plays a critical role in regulating cellular level of H_2_O_2_^14^,^53^. Consequently, further work will be directed to compare if the guard cells of *TaGLDH-A1a* and *TaGLDH-A1b* leaves may differ in the levels of AsA and H_2_O_2_ under normal or water deficiency conditions.

Because it has been shown that GLDH regulates the assembly and function of plant RCI^10^,^15^, it will also be interesting to investigate if RCI activity may differ between *TaGLDH-A1a* and *TaGLDH-A1b* plants, and whether such difference might be involved in the enhanced drought tolerance associated with *TaGLDH-A1b*. Two recent studies have shown that partial reduction of RCI activity can lead to constitutive enhancement of drought tolerance in tobacco plants through reducing stomatal conductance and aperture^54^,^55^. Thus, it is worthy to analyze if the RCI activity of *TaGLDH-A1b* plants may be altered as compared to those carrying *TaGLDH-A1a*, and if so, its potential impact on stomatal aperture control and leaf water loss behavior.

Finally, owing to the difficulty in designing homoeolog specific primers, we were unable to compare if *TaGLDH-A1a* and *TaGLDH-A1b* may differ in their expression level by qRT-PCR. The resolving of this question is clearly important for further studying the functional difference between the two alleles. Therefore, we have started a RNA sequencing experiment, which should reveal any potential difference between the transcript levels of *TaGLDH-A1a* and *TaGLDH-A1b*. Because we observed a co-segregation between *TaGLDH-A1b* and reduced leaf water loss rate (Fig. 8B), and the occurrence of smaller stomatal aperture in *TaGLDH-A1b* plants (Fig. 9), it becomes necessary to examine if *TaGLDH-A1b* may co-segregate with decreased stomatal aperture. However, the investigation of this question using segregating F_2_ plants is time consuming. The development of defined mutants for *TaGLDH* homoeologs (see above) or near isogenic lines for *TaGLDH-A1a* and *TaGLDH-A1b* should facilitate this analysis in further research.

In summary, this work has improved our understanding on plant *GLDH* genes and their function through characterizing *TaGLDH* homoeologs and discovering an association between *TaGLDH-A1b* and enhanced drought tolerance. The new information obtained may prompt further research on *GLDH* genes and their application in improving the drought tolerance and other traits of crop plants.

## Methods

### Plant materials, nucleic acid extraction, oligonucleotide primers and chemicals

Four sets of common wheat varieties were used in this study. The first set included Xiaoyan 54, CS and the NT lines derived from CS. The second set was the mini-core collection (MCC) of Chinese wheat germplasm composed of 262 landraces and improved varieties^27^. The third set included 11 cultivars from Xinjiang (Table S2). The fourth set consisted of 134 F_2_ seedlings developed in this work through crossing two Xinjiang varieties (i.e., Yichun 4 × Xinchun 3). Unless specifically stated, wheat plants were grown in a greenhouse at 25 °C (day)/20 °C (night) and with a 16 h light/8 h dark photoperiod. A vernalization treatment (5 weeks at 4 °C) was applied to Xiaoyan 54 seedlings to promote flowering. Genomic DNA and total RNA samples were prepared from desired wheat tissues as described previously^40^. The oligonucleotide primer sets (PS1 to PS11) used in this work are listed in Table S1, and their positions are given in Figure S1.

### Cloning *TaGLDH* homoeologs and expression analysis of *TaGLDH*

The BAC library of Xiaoyan 54 was screened by PCR with the conserved primer set PS1 (Table S1, Figure S1), and following the procedure reported previously^56^. *TaGLDH* ORFs, corresponding to *TaGLDH-A1*, *-B1* and *-D1*, respectively, were amplified from the positive BAC clones using the primer set PS2 (Table S1, Figure S1). PS2 was also used to clone the cDNA coding region of *TaGLDH-A1*, *-B1* and *-D1* by RT-PCR with the cDNAs derived from Xiaoyan 54 leaf tissues as described previously^40^. Subsequently, two primer sets (PS3 and PS4, Table S1, Figure S1), both of which could yield amplicons with different lengths for *TaGLDH-A1*, *-B1* and *-D1*, were developed for chromosome assignment of the three homoeologs. The assignment was facilitated using CS and associated NT lines and by fragment analysis of fluorescently labeled PCR amplicons^57^.

Because the coding sequences of *TaGLDH-A1*, *-B1* and *-D1* were nearly identical, it was difficult to design copy specific PCR primers to study the expression patterns of individual homoeologs. Therefore, two primer sets PS5 and PS6 (Table S1, Figure S1), capable of recognizing all three *TaGLDH* homoeologs, were designed for detecting the transcript level of *TaGLDH* by qRT-PCR and semi-quantitative RT-PCR, respectively. Total RNA samples were extracted from vegetative (root, stem, seedling leaf and flag leaf) and reproductive (young and mature spikelets) organs. The cDNAs derived from the different organs were used for qRT-PCR with the 26S rRNA gene (GenBank accession Z11889) as an internal reference and following the cycling conditions reported previously^40^. The semi-quantitative RT-PCR assay was performed as reported in a previous study^57^, with the wheat beta-tubulin gene 2 (GenBank accession U76745) as an internal reference gene.

### Ectopic expression of TaGLDH homoeologs in *N. benthamiana*

The coding sequence of *TaGLDH-A1*, *-B1* or *-D1* was each expressed in *N. benthamiana* using the PEBV vector. This vector has been used successfully for ectopically expressing cloned genes in *N. benthamiana*^58^,^59^. For constructing PEBV:GUS, GUS coding sequence was excised from the plasmid pJIT166^60^ using *Nco*I and *Eco*RI digestions, followed by cloning into PEBV vector. The coding sequence of *TaGLDH-A1*, *-B1* and *-D1* was each cloned into PEBV vector using PCR fragment amplified with the primer sets PS7 (for *A1*) or PS8 (for *B1* and *D1*) to form PEBV:A1, PEBV:B1 and PEBV:D1, respectively. The information on PS7 and PS8 is given in Table S1 and Figure S1. The inoculation of *N. benthamiana* plants with PEBV:GUS, PEBV:A1, PEBV:B1 or PEBV:D1 was conducted as detailed in our previous publication^59^. Two weeks after inoculation, the leaves infected by PEBV:GUS were collected for histochemical staining of GUS activity^61^, whereas those infected by PEBV:A1, PEBV:B1 or PEBV:D1 were harvested for determining total GLDH activity and AsA content (see below).

### VIGS analysis

Among the three recombinant BSMVs used in this analysis (Figure S4a), BSMV:GFP and BSMV:PDSas were developed previously^40^,^42^. BSMV:GLDHas was constructed using a 431 bp sequence element conserved among *TaGLDH-A1*, *-B1* and *-D1* coding sequence. This element was amplified from leaf cDNAs as a *Nhe*I fragment using the primer set PS9 (Table S1, Figure S1). It was cloned into BSMV vector and gave rise to BSMV:GLDHas. The genome wide target specificity of the 431 bp fragment was checked by searching the draft genome sequence of CS at http://plants.ensembl.org/Triticum_aestivum/Info/Index.

RNA transcripts were prepared for the three recombinant BSMVs, and introduced into Xiaoyan 54 seedlings (at two leaf stage) as detailed previously^40^,^42^. For each virus, 60 seedlings were inoculated. The same number of seedlings was buffer-inoculated as mock controls, and another 60 seedlings were kept as uninoculated controls. Three weeks after inoculation, the fourth leaves of the plants infected by BSMV:GLDHas were collected for examining *TaGLDH* silencing by qRT-PCR and alterations of total GLDH activity and AsA content (see below). For testing the effect of exogenous application of L-GalL on AsA biosynthesis, the fourth leaves were collected from mock controls and the plants infected by BSMV:GFP or BSMV:PDSas. They were cut into 1 mm segments, followed by incubation in a 15 mM solution of L-GalL for 24 h at 25 °C under a light intensity of 150 μmol/m^2^·s. Afterwards, the segments were collected for measuring total and reduced AsA contents (see below).

### Measurement of total GLDH activity and AsA content in leaf tissues

The assay of total GLDH activity in leaf tissues was carried out using a crude mitochondrial fraction that was prepared at 4 °C following a previous study^11^. Measurement of GLDH activity level in the crude mitochondrial fraction was conducted in 0.5 ml volume consisting of 50 mM Tris-HCl, pH 8.0, 60 μM cytochrome c, 0.15% (w/v) Triton X-100 and 25 μg sample protein. The reaction was started by adding 4 μM L-GalL. After the reaction, the assay mixtures (150 μl per sample) were transferred to a 96 well microtiter plate, and the absorbance at 550 nm was recorded for each well using a Thermo Multiscan Scientific Spectrophotometer (Thermo Scientific, Waltham, USA). One unit of GLDH activity was defined as the formation of 2 nm reduced cytochrome c per minute.

To determine AsA content in a leaf sample, about 40 mg foliar materials were homogenized, and suspended in 6% trichloroacetic acid. The suspension was centrifuged at 13,000 g for 5 min at 4 °C, and the supernatant was kept for measuring the contents of total and reduced AsA following the method detailed previously^62^.

### Screening of *TaGLDH* variants and tagging of *TaGLDH-A1b*

The screening was executed by fragment analysis of fluorescently labeled PCR amplicons. Briefly, genomic DNA samples were extracted from 262 MCC lines, and used in two series of genomic PCR assays with the primer sets PS3 and PS4 (Table S1, Figure S1), respectively. The resultant amplicons were subjected to fragment analysis as described above. For tagging *TaGLDH-A1b* allele, a cleaved amplified polymorphic marker CAP-A1b was developed (Figure S7a). A portion of the genomic ORF of *TaGLDH-A1* (538 bp for *TaGLDH-A1a* and 535 bp for *TaGLDH-A1b*) was specifically amplified by PCR with the primer set PS11 (Table S1, Figure S1). The amplicons were digested with *Bsa*JI, which cut the amplicons from *TaGLDH-A1a* but not those of *TaGLDH-A1b*. CAP-A1b was employed to identify *TaGLDH-A1b* in 11 Xinjiang common wheat lines, and in the F_2_ individuals derived from Yichun 4 × Xinchun 3 (see below).

### Bacterial expression and biochemical study of TaGLDH-1a and -A1b

*TaGLDH-A1a* and *TaGLDH-A1b* sequences were amplified from the cDNAs of Xiaoyan 54 and Kashibaipi, respectively, using the primer set PS10 (Table S1, Figure S1). The resultant *TaGLDH-A1a* and *TaGLDH-A1b* fragments lacked the nucleotide sequence encoding the first 78 residues (the putative mitochondrial targeting peptide). These fragments were cloned into the plasmid vector pET-30a (Novagen, Madison, WI, USA), yielding the constructs pET-A1a and pET-A1b, respectively. Induction of TaGLDH-A1a and TaGLDH-A1b expression and purification of the two recombinant proteins by combining nickel chelate affinity chromatography and fast performance liquid chromatography followed the practices detailed previoulsy^59^. The purified TaGLDH-A1a and TaGLDH-A1b were checked by 10% SDS-PAGE and a protein blot assay with an anti-histidine antibody (Roche Diagnostic GmbH, Mannheim, Germany).

For investigating the kinetic properties of recombinant TaGLDH-A1a and TaGLDH-A1b, the assay was performed as described above for testing GLDH activity with minor modifications. Each assay was conducted in 0.2 ml volume consisting of 25 mM Tris-HCl, pH 8.0, 25 mM NaCl, 60 μM cytochrome c, 0.167 μg recombinant protein (TaGLDH-A1a or TaGLDH-A1b) and a specific concentration of L-GalL. A total of 11 concentrations of L-GalL, varying from 0.015625 to 5 mM, were used. The reaction was started by addition of TaGLDH-A1a or TaGLDH-A1b. After the reaction, the absorbance at 550 nm was read for each assay. The obtained readings were then employed to calculate the kinetic parameters (*K*_m_ and *V*_max_) with the SigmaPlot Enzyme Kinetics Module for Windows (SPSS Inc., Chicago, IL, USA).

### Drought tolerance growth trial and determination of free proline content

The trial was conducted essentially as described by Zhang and coauthors^33^. In brief, each trail started with the seedlings at three-leaf stage, and a total of 40 uniform seedlings (5 individuals per pot) were used for each variety. The plants were placed in a growth chamber set at 23 °C (day)/20 °C (night) and with a 16 h light/8 h dark photoperiod, but without watering for 30 days. They were re-watered afterwards. The volumetric water content (VWC) of each pot was checked every two days, and the plants at the same VWC were used for comparison. The trial was repeated three times. At 20 days post water deprivation, free proline content was determined for each variety using the leaves collected from 5 individual plants in five different pots. The measurement of proline content followed a procedure detailed previoulsy^63^. The proline content assay was conducted in two of the three drought tolerance trails.

### Water loss assay

The assay was carried out using wheat plants (each with 3 tillers) cultured in 1 × Hoagland solution in a growth chamber with a temperature regime of 15 °C (day)/10 °C (night) and a photoperiod of 16 h light/8 h dark. In each assay and for each cultivar, 7 fully expanded leaves from 7 plants were pooled and subjected to water loss measurement^33^. For investigating water loss behavior of the F_2_ plants derived from Yichun 4 × Xinchun 3, the F_2_ individuals (at tillering stage) were first genotyped with the marker CAP-A1b (see above). This divided the F_2_ progenies into three groups, homozygous for *TaGLDH-A1a* (i.e., *A1aA1a*) or *TaGLDH-A1b* (*A1bA1b*) and heterozygous at *TaGLDH-A1* locus (*A1aA1b*) (Figure S7b). The water loss assay was then conducted for the three types of plants and their parents as described above.

### Measurement of stomatal aperture

Wheat plants were cultured as described above for the water loss assay. For each of the eight wheat varieties under examination, 9 uniform plants (each with 3 tillers) were selected and divided into 3 batches (3 in each batch). The first batch of plants was transferred into double distilled water (as controls), with 6 epidermal peels immediately prepared from the 3 plants. The second and third batches of plants were transferred into 13% PEG 6000, and treated for 24 and 48 h, respectively. Subsequently, 6 epidermal peels were immediately prepared from the second and third batches of plants, respectively. The epidermal peels were stained with 2′,7′-dichlorofluorescin diacetate^64^, which facilitated the examination of guard cells and stomatal aperture under a confocal laser scanning microscope (LSM710; Carl Zeiss AG, Oberkochen, Germany). Ten photos were taken for each epidermal peel, and used for measuring stomatal aperture with the ZEN lite 2012 software. This experiment was repeated three times.

### Statistical analysis

Statistical analysis of the experimental data (presented as mean ± SD) was performed by ANOVA with the SPSS program (SAS Institute Inc., Cary, NC, USA).

## Additional Information

**Accession codes:** The genomic sequences of *TaGLDH-A1*, *-B1* and *-D1* have been deposited in the GenBank under the accession codes KU695146, KU695147 and KU695148, respectively.

**How to cite this article**: Zhang, J. *et al.* A novel allele of L-galactono-1,4-lactone dehydrogenase is associated with enhanced drought tolerance through affecting stomatal aperture in common wheat. *Sci. Rep.* **6**, 30177; doi: 10.1038/srep30177 (2016).

## Supplementary Material

Supplementary Information

## Figures and Tables

**Table 1:** Kinetic parameters of recombinant TaGLDH-A1a and -A1b proteins^a^.

Recombinant protein	*K*_m_ (μM)	*V*_max_ (μmol.mg^−1^protein.min^−1^)
TaGLDH-A1a	61.0 ± 5.4	11.43 ± 0.88
TaGLDH-A1b	181.0 ± 67.1*	0.16 ± 0.04***

^a^Values are means ± SD of three independent assays. Statistical analysis between the Km or Vmax values was conducted with ANOVA. *Indicates significant difference from the Km of TaGLDH-A1a at *P* < 0.05. ^***^Indicates significant difference from the *V*_max_ of TaGLDH-A1a at *P* < 0.001.
