# Supplementary material for: A novel allele of L-galactono-1,4-lactone dehydrogenase is associated with enhanced drought tolerance through affecting stomatal aperture in common wheat
Source: Sci Rep. 2016 Jul 22;6:30177. doi: 10.1038/srep30177 (PMC4957090; doi:10.1038/srep30177)
Supplement: Supplementary Information [file srep30177-s1.pdf]

A novel allele of L-galactono-1,4-lactone dehydrogenase is associated with enhanced drought tolerance through affecting stomatal aperture in common wheat

Juncheng Zhang[1,+], Bin Li[1,2,3,+], Yanping Yang[1,2], Peiyuan Mu[4], Weiqiang Qian[1,2], Lingli Dong[1], Kunpu Zhang[1], Xin Liu[1], Huanju Qin[1], Hongqing Ling[1] & Daowen Wang[1,5*]

[1]The State Key Laboratory of Plant Cell and Chromosome Engineering, Institute of Genetics and Developmental Biology, Chinese Academy of Sciences, Beijing 100101, China. [2]University of Chinese Academy of Sciences, Beijing 100049, China. [3]The National Key Facility for Crop Gene Resources and Genetic Improvement, Institute of Crop Science, Chinese Academy of Agricultural Sciences, Beijing 100081, China. [4]Institute of Crop Research, Xinjiang Academy of Agri-Reclamation Sciences, Shihezi 832000, China. [5]The Collaborative Innovation Center for Grain Crops, Henan Agricultural University, Zhengzhou 450002, China.

[+]The two authors contributed equally to this work.

Correspondence and request for materials should be addressed to D.W. (email dwwang@genetics.ac.cn).

Figure S1. A diagram showing the positions of the different primer sets (PS1 to PS11) used in this work. The positions are relative to the genomic open reading frame of *TaGLDH-A1* (from start to stop codons). The green boxes represent exons. The nucleotide sequences of the primers are listed in Table S1.

Figure S2. Assigning *TaGLDH-A1*, *-B1* and *-D1* to wheat group 5 chromosomes 5A, 5B and 5D, respectively, through comparing the amplicons obtained with the primer sets PS3 or PS4 in Chinese Spring (CS) and derivative nulli-tetrasomic (NT) lines (N5AT5D, N5BT5A and N5DT5B). The amplicons (peaks) derived from *TaGLDH-A1*, *-B1* and *-D1* were separated by capillary electrophoresis. Those missed in the individual NT lines are indicated by arrows.

Figure S3. Recombinant pea early browning viruses (PEBVs) developed (a) and β-glucuronidase (GUS) signals generated by the infection of *N. benthamiana* plants with PEBV:GUS (b). Four T-DNA constructs (pCAPE2-GUS, pCAPE2-A1, pCAPE2-B1 and pCAPE2-D1) were prepared. Together with pCAPE1, they formed four recombinant PEBVs (PEBV:GUS, PEBV:A1, PEBV:B1 and PEBV:D1), respectively. PEBV:GUS was introduced into *N. benthamiana* plants through agroinfection, with the ectopically expressed GUS protein visualized by histochemical staining at two weeks post agroinfection. The blue precipitates in (b) indicate GUS protein expression. CP, coat protein; LB, left border; RB, right border; T, NOS terminator; 35S, 35S promoter.

Figure S4. Recombinant barley stripe mosaic viruses (BSMVs) developed (a), green florescence protein (GFP) signals produced by BSMV:GFP (b), and leaf bleaching phenotype generated by BSMV:PDSas (c). Three γ clones (γ-GFP, γ-TaPDSas and γ-TaGLDHas) were used in this work. When combined with the α and β clones, three

recombinant BSMVs (BSMV:GFP, BSMV: PDSas and BSMV:GLDHas) were reconstituted. At two weeks after virus inoculation of Xiaoyan 54 seedlings, GFP signals were detected in the leaf tissues infected by BSMV:GFP but not in those of uninoculated control under fluorescent microscopy; strong photo-bleaching was found in the leaves infected by BSMV:PDSas but not in those of uninoculated control or infected by BSMV:GFP. The two *Nhe*I restriction enzyme digestion sites were used to clone foreign insert into the original γ clone. αa, βa, βb, βc, βd, γa and γb are BSMV open reading frames.

Figure S5. Stimulation of AsA biosynthesis by exogenous supply of 15 mM L-galactono-1,4-lactone (L-GalL). The leaves collected from mock controls and the plants infected by BSMV:GFP or BSMV:GLDHas were treated with 15 mM L-GalL or distilled water (dd H_2O), followed by measurement of total AsA (a) and reduced AsA (b) contents. Each value (± SD) is the mean of three independent measurements (for three plants). The values marked by different letters are statistically significant ($P \leq 0.05$).

Figure S6. Detection of *TaGLDH-A1a* and *TaGLDH-A1b* alleles in common wheat germplasm lines by examining PCR fragments amplified with the primer sets PS3 or PS4. *TaGLDH-A1a* was detected in Xiaoyan 54 and Chinese Spring (CS), whereas *TaGLDH-A1b* was found in Hongdongmai, Kashi 1, Kashibaipi and Tutoumai. The fragment derived from *TaGLDH-A1b* (452 bp) was 3 bp shorter than that from *TaGLDH-A1a* (455 bp). The six varieties did not differ in the size of the remaining five fragments.

Figure S7. Development and application of a cleaved amplified polymorphic marker CAP-A1b for differentiating *TaGLDH-A1b* from its wild type allele *TaGLDH-A1a*. (a)

The polymorphism pattern generated by CAP-A1b with the PCR amplicons from two varieties (Kashibaipi and Xinchun 3) carrying *TaGLDH-A1b* and another two lines (Xiaoyan 54 and Yichun 4) with *TaGLDH-A1a*. After digestion with *Bsa*JI, the amplicons of *TaGLDH-A1a* were cleaved into two fragments of 266 and 272 bp, respectively, whereas those of *TaGLDH-A1b* remained intact. The size (bp) of the DNA marker is shown on the left side of the graph. (b) Application of CAP-A1b in genotyping the individuals in a F_2 population prepared by crossing Yichun 4 (*TaGLDH-A1a*) with Xinchun 3 (*TaGLDH-A1b*). This marker allowed the identification of the F_2 individuals homozygous for *TaGLDH-A1a* (blue) or *TaGLDH-A1b* (red) and those segregating for *TaGLDH-A1a* and *TaGLDH-A1b* (green).

Figure S8. AsA contents in the leaf (a) and root (b) tissues of the wheat landrace Kashibaipi (carrying the allele *TaGLDH-A1b*) before and after water deficiency treatment using 13% polyethylene glycol 6000 (PEG 6000). The plants used in this test were three weeks old. After being subjected to PEG 6000 treatment for the indicated times, the leaf and root samples were collected for measuring the contents of reduced AsA and oxidized AsA. Each value (± SD) was the mean of three independent measurements (using the samples from three different plants).

Table S1. Primer sets used in this study.

Table S2. List of 11 common wheat cultivars from Xinjiang and their allelic status at *TaGLDH-A1* locus.

Table S3. Abiotic stress tolerance, cultivation history and pedigree (origin) information for the seven common wheat cultivars carrying *TaGLDH-A1b*.

Figure S1

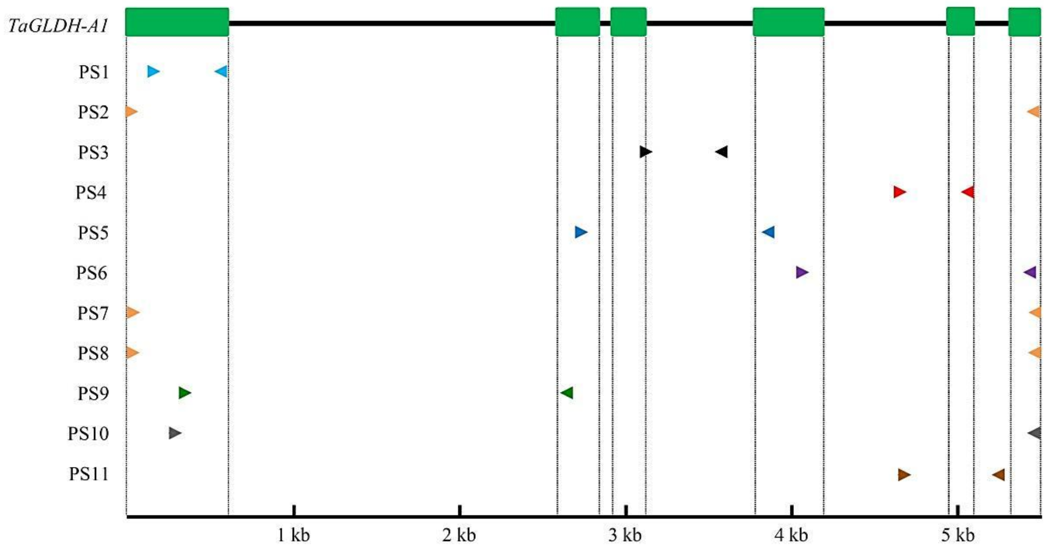

TaGLDH-A1
PS1
PS2
PS3
PS4
PS5
PS6
PS7
PS8
PS9
PS10
PS11
1 kb
2 kb
3 kb
4 kb
5 kb

Figure S2

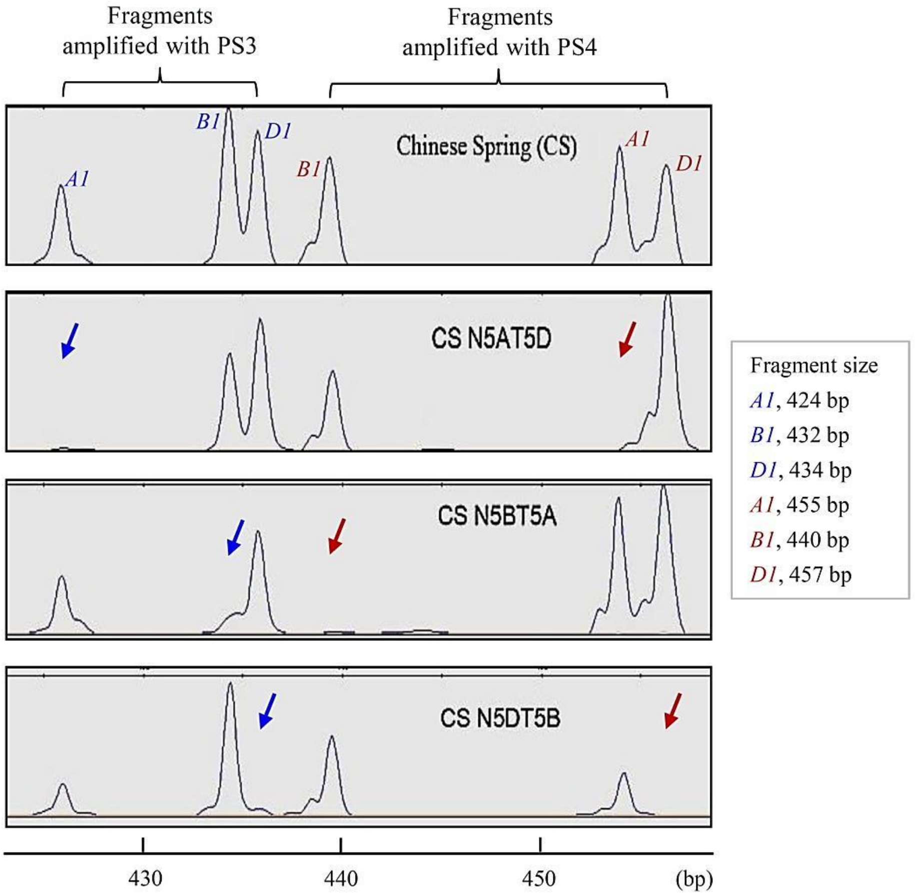

Fragments amplified with PS3
Fragments amplified with PS4
Chinese Spring (CS)
A1
B1
D1
B1
A1
D1
CS N5AT5D
CS N5BT5A
CS N5DT5B
430
440
450
(bp)
Fragment size
A1, 424 bp
B1, 432 bp
D1, 434 bp
A1, 455 bp
B1, 440 bp
D1, 457 bp

Figure S3

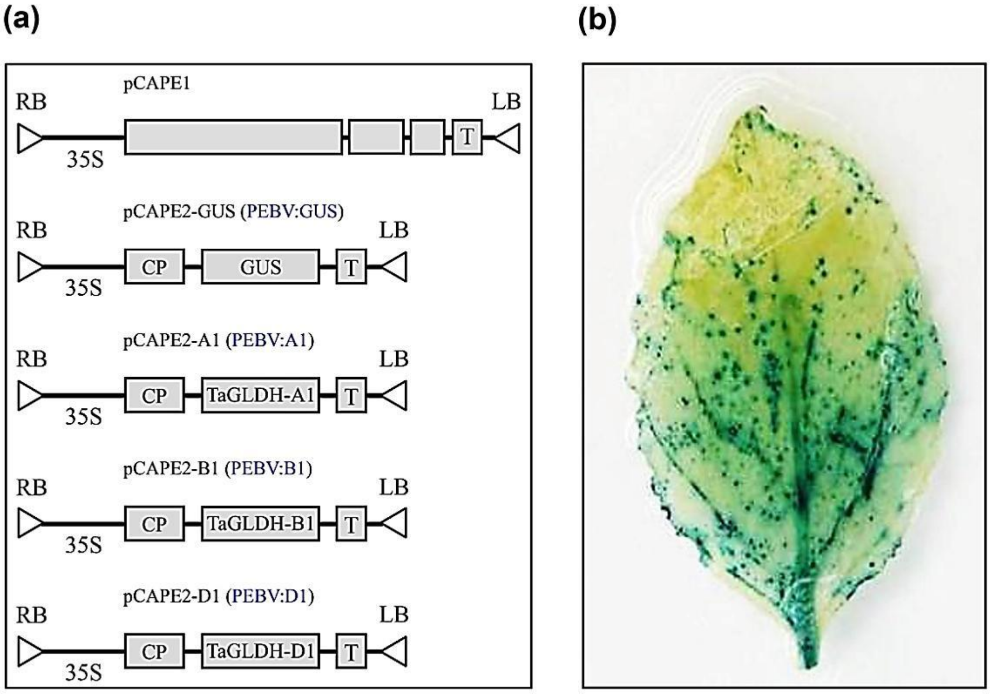
(a)
(b)
pCAPE1
RB
LB
35S
T
pCAPE2-GUS (PEBV:GUS)
CP
GUS
pCAPE2-A1 (PEBV:A1)
TaGLDH-A1
pCAPE2-B1 (PEBV:B1)
TaGLDH-B1
pCAPE2-D1 (PEBV:D1)
TaGLDH-D1

Figure S4

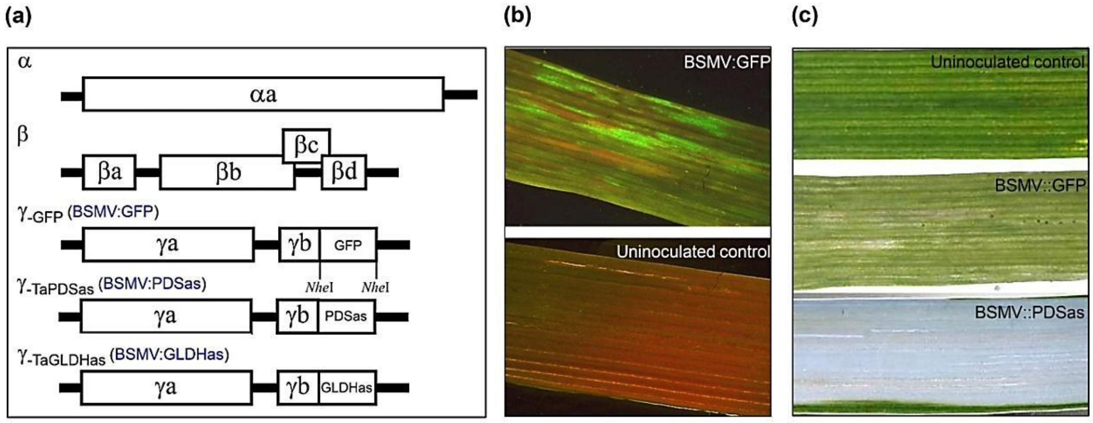
(a)
(b)
(c)
α
αa
β
βa
βb
βc
βd
γ-GFP (BSMV:GFP)
γa
γb
GFP
NheI
NheI
γ-TaPDSas (BSMV:PDSas)
γa
γb
PDSas
γ-TaGLDHas (BSMV:GLDHas)
γa
γb
GLDHas
BSMV:GFP
Uninoculated control
Uninoculated control
BSMV::GFP
BSMV::PDSas

Figure S5

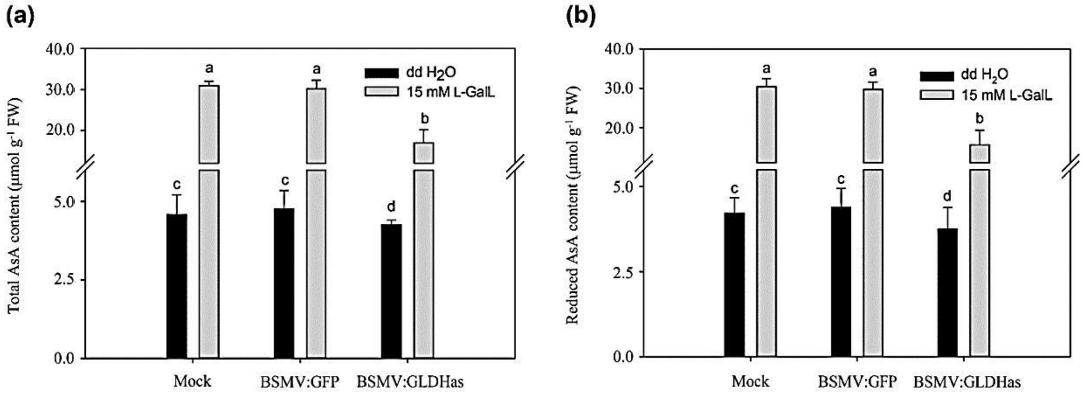
(a)
40.0
30.0
20.0
5.0
2.5
0.0
Total AsA content (µmol g-1 FW)
a
a
b
c
c
d
dd H2O
15 mM L-GalL
Mock
BSMV:GFP
BSMV:GLDHas
(b)
40.0
30.0
20.0
5.0
2.5
0.0
Reduced AsA content (µmol g-1 FW)
a
a
b
c
c
d
dd H2O
15 mM L-GalL
Mock
BSMV:GFP
BSMV:GLDHas

Figure S6

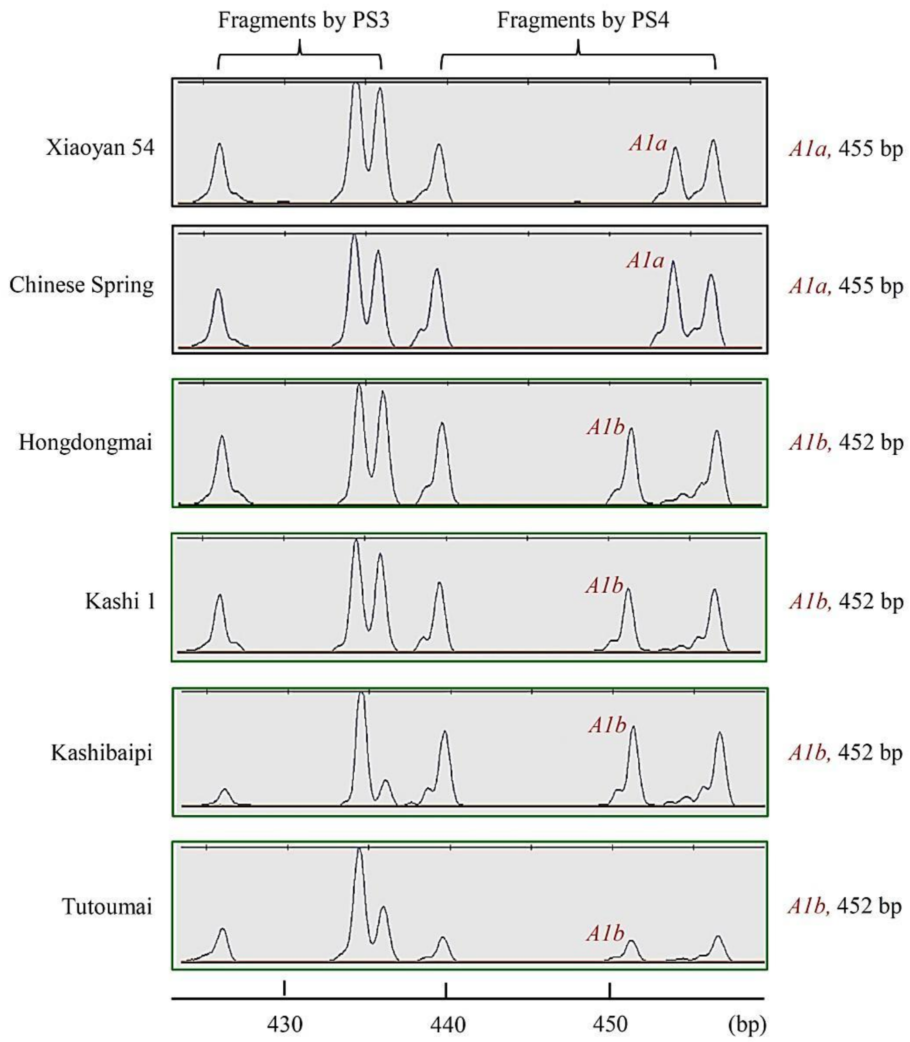

Fragments by PS3
Fragments by PS4
Xiaoyan 54
A1a
A1a, 455 bp
Chinese Spring
A1a
A1a, 455 bp
Hongdongmai
A1b
A1b, 452 bp
Kashi 1
A1b
A1b, 452 bp
Kashibaipi
A1b
A1b, 452 bp
Tutoumai
A1b
A1b, 452 bp
430
440
450
(bp)

Figure S7

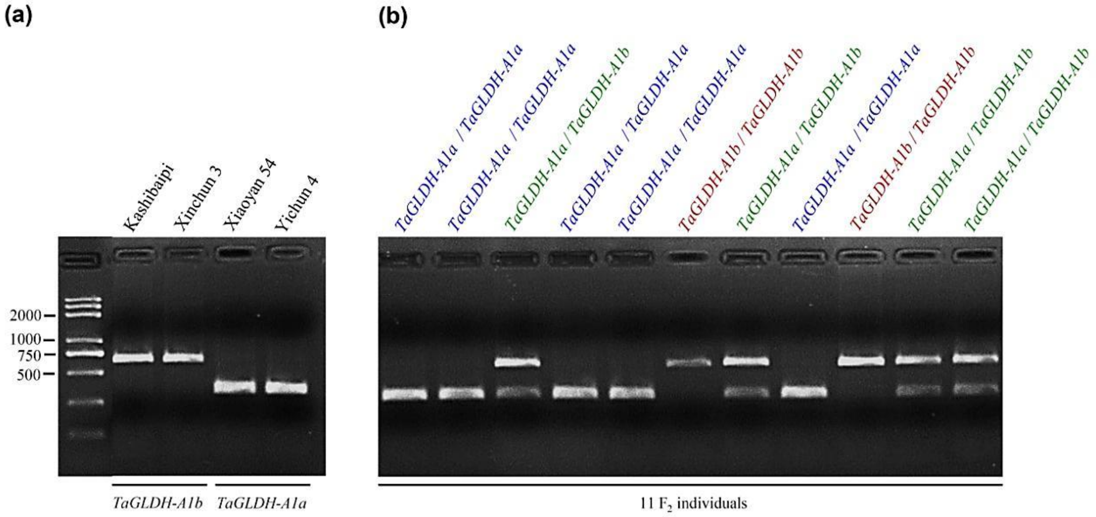
(a)
Kashibaipi
Xinchun 3
Xiaoyan 54
Yichun 4
2000
1000
750
500
TaGLDH-A1b
TaGLDH-A1a
(b)
TaGLDH-A1a / TaGLDH-A1a
TaGLDH-A1a / TaGLDH-A1a
TaGLDH-A1a / TaGLDH-A1b
TaGLDH-A1a / TaGLDH-A1a
TaGLDH-A1a / TaGLDH-A1a
TaGLDH-A1b / TaGLDH-A1b
TaGLDH-A1a / TaGLDH-A1b
TaGLDH-A1a / TaGLDH-A1a
TaGLDH-A1b / TaGLDH-A1b
TaGLDH-A1a / TaGLDH-A1b
TaGLDH-A1a / TaGLDH-A1b
11 F2 individuals

Figure S8

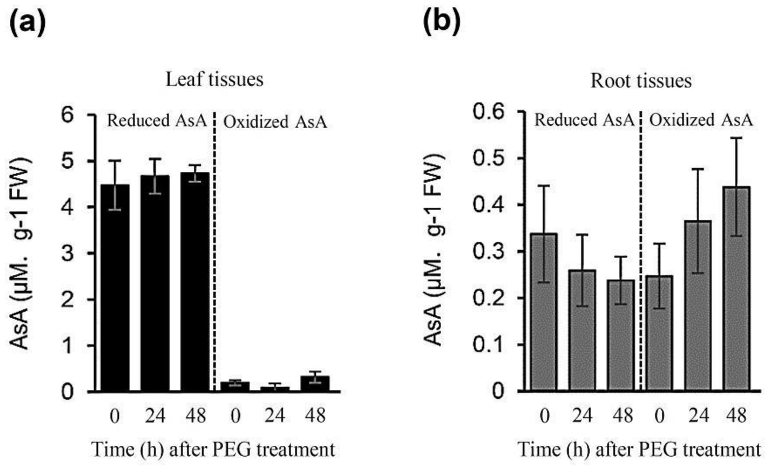

(a)
Leaf tissues
Reduced AsA
Oxidized AsA
AsA (μM. g-1 FW)
6
5
4
3
2
1
0
0 24 48 0 24 48
Time (h) after PEG treatment
(b)
Root tissues
Reduced AsA
Oxidized AsA
AsA (μM. g-1 FW)
0.6
0.5
0.4
0.3
0.2
0.1
0
0 24 48 0 24 48
Time (h) after PEG treatment

Table S1. Primer sets used in this study.

Primer set	Sequence (5′ - 3′) [a]	Amplicon length (bp)	Use
PS1	F: TCCACAAGAAGGCCGTCCCCTTC<br>R: ACCTGAATGATGCCGCCGACCTGC	378<br>(Genomic DNA)	Screening Xiaoyan 54 BAC library
PS2	F: ATGCGGCACCTCCTCCTCTCCCGTC<br>R: TTACC(T)TTGCATGTTGGACAGTCTG	5498 - 5685<br>(Genomic DNA)<br><br>1755 bp (cDNA)	1) Amplifying full-length *TaGLDH* ORFs from positive BAC clones<br>2) Cloning the cDNA coding region of *TaGLDH-A1*, *-B1* and *-D1*
PS3	F: GAGTCATTGAAGAAATATAGGTGCG<br>R: GCGACTCTTCCTACAAACATCAC	424 (*A1*) [b]<br>432 (*B1*)<br>434 (*D1*)<br>(Genomic DNA)	Assigning *TaGLDH* homoeologs to wheat chromosomes
PS4	F: CCCTGTATAACTCAGTTCAAACTGTG<br>R: GCAGAATAATCATCCCAGAGACTAG	455 (*A1*)<br>440 (*B1*)<br>457 (*D1*)<br>(Genomic DNA)	Assigning *TaGLDH* homoeologs to wheat chromosomes
PS5	F: CTTGCC(T)CGCTGTGGACTTG<br>R: TCTATCGCTGGGTCTTTACTGTCG	341 (cDNA)	Measuring *TaGLDH* expression by qRT-PCR
PS6	F: CCTACAGGAACTCTAGCGAAGC<br>R: AACTTGGCACTGGAGAGAACC	446 (cDNA)	Measuring *TaGLDH* expression by semi-quantitative RT-PCR
PS7	F: CTGAGCTCATGCGGCACCTCCTCCTC<br>R: CTGTCGACTTACCTTGCATGTTGGAC	1755 (cDNA)	Cloning *TaGLDH-A1* into PEBV vector
PS8	F: CTGAGCTCATGCGGCACCTCCTCCTC<br>R: CTGTCGACTTACTTTGCATGTTGGAC	1755 (cDNA)	Cloning *TaGLDH-B1* or *D1* into PEBV vector
PS9	F: CTGCTAGCCCACACCGTCTCCAACTG<br>R:CTGCTAGCCTTTGGCAGGAGTAACCAG	431 (cDNA)	Constructing BSMV:GLDHas for VIGS
PS10	F: GTCATATGTATGCACCTCTTCCTGACGACCTCCACAC [c]<br>R: CTGCGGCCGCCTTTGCATGTTGGACAGTC	1521 (cDNA)	Expressing TaGLDH-A1a and -A1b in bacterial cells
PS11	F: AGTATTGGTAATAAATTAACAAC<br>R: CCATTTTGTCAATAATGCTTT	538 bp (*TaGLDH-A1a*)<br>535 bp (*TaGLDH-A1b*)<br>(Genomic DNA)	Development of the CAP-A1b marker for identifying *TaGLDH-A1b*
PS12 (Wheat 26S rRNA gene) [d]	F: GAAGAAGGTCCCAAGGGTTC<br>R: TCTCCCTTTAACACCAACGG	110 (cDNA)	As internal reference for qRT-PCR
PS13 (Wheat beta-tubulin gene 2) [e]	F: GTGCATGGTTCTTGACAACG<br>R: CCTTAGGCAGCAGGTGACTC	130 (cDNA)	As internal reference for semi-quantitative RT-PCR

[a] The underlined nucleotides form the restriction enzyme digestion site for *Sac*I (GAGCTC), *Sal*I (GTCGAC), *Nde*I (CATATG), *Nhe*I (GCTAGC) or *Not*I (GCGGCCGC).

[b] *A1*, *B1* and *D1* denote *TaGLDH-A1*, *-B1* and *-D1* homoeologs, respectively.

[c] In this forward primer, the sequence downstream of the underlined ATG (the start codon for bacterial expression), i.e., TATGCACCTCTTCCTGACGACCTCCACAC, differed slightly from its target region in the coding sequence of TaGLDH-A1a and A1b, but still encoded the correct amino acid residues (YAPLPDDLH) upon translation. This modification reduced the GC content and facilitated the expression of TaGLDH-A1a and A1b in bacterial cells.

[d] The GenBank accession number for the wheat 26S rRNA gene is Z11889.

[e] The GenBank accession number for the wheat beta-tubulin gene 2 is U76745.

Table S2. List of 11 common wheat cultivars from Xinjiang and their allelic status at *TaGLDH-A1* locus.

Cultivar	*TaGLDH-A1* allele
Achun 2	*TaGLDH-A1a*
Changchun 3	*TaGLDH-A1a*
Dabaimai	*TaGLDH-A1a*
Jiuchun 1	*TaGLDH-A1b*
Jiuchun 2	*TaGLDH-A1a*
Qichun 5	*TaGLDH-A1a*
Redstar	*TaGLDH-A1b*
Tuchun 6	*TaGLDH-A1a*
Xinchun 2	*TaGLDH-A1a*
Xinchun 3	*TaGLDH-A1b*
Yichun 4	*TaGLDH-A1a*

Table S3. Abiotic stress tolerance, cultivation history and pedigree (origin) information for the seven common wheat cultivars carrying *TaGLDH-A1b*.

Cultivar	Type	Abiotic stress tolerance	Cultivation history	Pedigree (origin)
Hongdongmai	Landrace	Tolerance to drought, hot wind, salt and cold stresses	1950s	Local line
Kashi 1	Improved variety	Tolerance to drought, salt and cold stresses	1970s	Bacpuc × Heine Hvede
Kashibaipi	Landrace	Tolerance to drought, hot wind and salt stresses	1950s - 1970s	Local line
Redstar	Landrace	Tolerance to drought and hot wind stresses	1960s - early 1980s	From former Soviet Union
Totoumai	Landrace	Tolerance to drought and hot wind stresses	1950s - 1960s	Local line
Jiuchun 1	Improved variety	Tolerance to drought and hot wind stresses	1980s	Honglema × 5-1-3
Xinchun 3	Improved variety	Tolerance to drought stress	1980s - 1990s	Siete Cerros × Changchun 1 (Changchun 1 is a derivative of Kashibaipi)

The information in this Table is derived from the following references.

Jin, S. B. *Chinese wheat varieties and their pedigrees*. (Beijing Agricultural Press, 1983).

Jin, S. B. *Chinese wheat varieties (1983-1993)*. (Beijing Agricultural Press, 1997).

He, Z. H., Rajaram, S., Xin, Z. Y. & Huang, G. Z. *A history of wheat breeding in China*. (CIMMYT, 2001).

Zhuang, Q. S. *Chinese wheat improvement and pedigree analysis*. (Beijing Agricultural Press, 2003).
